# A SelB/EF-Tu/aIF2γ-like protein from *Methanosarcina mazei* in the GTP-bound form binds cysteinyl-tRNA^Cys^

**DOI:** 10.1007/s10969-015-9193-6

**Published:** 2015-01-25

**Authors:** Tatsuo Yanagisawa, Ryohei Ishii, Yasushi Hikida, Ryuya Fukunaga, Toru Sengoku, Shun-ichi Sekine, Shigeyuki Yokoyama

**Affiliations:** 1 RIKEN Systems and Structural Biology Center, 1-7-22 Suehiro-cho, Tsurumi, Yokohama, 230-0045 Japan; 2 RIKEN Structural Biology Laboratory, 1-7-22 Suehiro-cho, Tsurumi, Yokohama, 230-0045 Japan; 3 RIKEN Center for Life Science Technologies, 1-7-22 Suehiro-cho, Tsurumi, Yokohama, 230-0045 Japan; 4 Department of Biophysics and Biochemistry, Graduate School of Science, The University of Tokyo, 7-3-1 Hongo, Bunkyo-ku, Tokyo, 113-0033 Japan; 5 Present Address: Department of Biochemistry, School of Medicine, Johns Hopkins University, 725 N. Wolfe Street, 521A Physiology Bldg., Baltimore, MD 21205 USA

**Keywords:** Crystal structure, Translation factor, GTP, tRNA

## Abstract

The putative translation elongation factor Mbar_A0971 from the methanogenic archaeon *Methanosarcina barkeri* was proposed to be the pyrrolysine-specific paralogue of EF-Tu (“EF-Pyl”). In the present study, the crystal structures of its homologue from *Methanosarcina mazei* (MM1309) were determined in the GMPPNP-bound, GDP-bound, and apo forms, by the single-wavelength anomalous dispersion phasing method. The three MM1309 structures are quite similar (r.m.s.d. < 0.1 Å). The three domains, corresponding to domains 1, 2, and 3 of EF-Tu/SelB/aIF2γ, are packed against one another to form a closed architecture. The MM1309 structures resemble those of bacterial/archaeal SelB, bacterial EF-Tu in the GTP-bound form, and archaeal initiation factor aIF2γ, in this order. The GMPPNP and GDP molecules are visible in their co-crystal structures. Isothermal titration calorimetry measurements of MM1309·GTP·Mg^2+^, MM1309·GDP·Mg^2+^, and MM1309·GMPPNP·Mg^2+^ provided dissociation constants of 0.43, 26.2, and 222.2 μM, respectively. Therefore, the affinities of MM1309 for GTP and GDP are similar to those of SelB rather than those of EF-Tu. Furthermore, the switch I and II regions of MM1309 are involved in domain–domain interactions, rather than nucleotide binding. The putative binding pocket for the aminoacyl moiety on MM1309 is too small to accommodate the pyrrolysyl moiety, based on a comparison of the present MM1309 structures with that of the EF-Tu·GMPPNP·aminoacyl-tRNA ternary complex. A hydrolysis protection assay revealed that MM1309 binds cysteinyl (Cys)-tRNA^Cys^ and protects the aminoacyl bond from non-enzymatic hydrolysis. Therefore, we propose that MM1309 functions as either a guardian protein that protects the Cys moiety from oxidation or an alternative translation factor for Cys-tRNA^Cys^.

## Introduction

GTP-binding translation factors play important roles in the initiation, elongation, and termination steps of translation. Translation elongation factor Tu (EF-Tu) (EF1α in eukaryotes/archaea), a GTP-binding translation factor, forms a complex with an aminoacyl-tRNA (aa-tRNA) and delivers it to the A site of the translating ribosome [reviewed in 1–6]. EF-Tu binds all canonical aa-tRNAs with nearly the same affinity, when each tRNA is bound to its cognate amino acid [7]. After correct codon-anticodon pairing, EF-Tu hydrolyzes the GTP, and the resultant EF-Tu·GDP complex dissociates from the aa-tRNA and the ribosome [8]. Thus, EF-Tu is responsible for the correct selection and binding of the cognate aa-tRNA to the codon at the A site. The translation elongation cycle is dependent on the different conformations of EF-Tu·GTP and EF-Tu·GDP [9–11].

Homologues of EF-Tu are also involved in the initiation of translation and/or the elongation cycle for non-canonical amino acids. In archaea and eukaryotes, the initiator Met-tRNA_i_ is delivered to the ribosome by initiation factor IF2. IF2 is a heterotrimeric complex in which the γ subunit, which is related to EF-Tu, binds GTP and Met-tRNA_i_ [12–14]. Another EF-Tu homologue protein, SelB, works as a special elongation factor for selenocysteine incorporation [15–17]. Selenocysteine is genetically encoded by an internal UGA stop codon and the specific mRNA stem-loop structure, called SECIS (selenocysteine insertion sequence) [18]. In bacteria, GTP-bound SelB recognizes and binds a selenocysteine-specific tRNA. Via its C-terminal domain (domain IV), this ternary complex subsequently binds SECIS in the ribosome-bound mRNA, resulting in the translational incorporation of selenocysteine in response to the specific internal UGA codons [19]. In mammals, the SelB homologue EF-Sec lacks domain IV, and the adaptor protein SPB2 binds EF-Sec and recognizes the SECIS element [20, 21].

By analogy to selenocysteine incorporation, a similar mechanism was proposed for pyrrolysine incorporation into proteins. Pyrrolysine is the “22nd” translationally inserted amino acid encoded by the UAG codon, and was first found in the monomethylamine methyltransferase (*mtmB1* gene product) from *Methanosarcina barkeri* [22–24]. Pyrrolysine is directly ligated to tRNA^Pyl^, bearing an anticodon complementary to the UAG codon, by pyrrolysyl-tRNA synthetase (PylRS) [25, 26]. In contrast to selenocysteine incorporation, the mechanism for the delivery of pyrrolysyl-tRNA^Pyl^ to the ribosome, and the decoding of the internal UAG codon as pyrrolysine, remain unclear. It was previously proposed that a specific elongation factor, EF-Pyl, is involved in pyrrolysine incorporation [27, 28].

All three known *Methanosarcina* genomes encode a protein homologous to SelB, EF-Tu, and aIF2γ [accession codes: WP_011033255 (Q8PXB3), WP_011305992 (Q46DU9), WP_011024522 (Q8TH68)], while no selenocysteine-containing proteins are encoded. Therefore, this SelB/EF-Tu/aIF2γ homologue was a candidate for EF-Pyl. However, the SelB/EF-Tu/aIF2γ-like proteins are shorter than the bacterial SelB proteins, and lack domain IV of SelB. Furthermore, no cis-acting elements corresponding to SECIS are conserved or functionally important for the genes encoding pyrrolysine-containing proteins [29, 30].

Numerous structures of the EF-Tu/SelB/aIF2γ superfamily proteins have been solved, including the GMPPNP-bound, GDP-bound, and apo form structures of the EF-Tu proteins from *Thermus thermophilus* [31, 32], *Thermus aquaticus* [33], and *Escherichia coli* [34–36], the aSelB from *Methanococcus maripaludis* [17], and the aIF2γ proteins from *Pyrococcus abyssi* [37], *Methanocaldococcus jannaschii* [38], *Pyrococcus furiosus* [39], and *Sulfolobus solfataricus* [12–14, 40, 41].

EF-Tu consists of three distinct domains, referred to as domains 1, 2, and 3. Domain 1 (the G domain) is responsible for guanine nucleotide binding, while domain 2 participates in tRNA and aminoacyl binding. All of the EF-Tu homologue structures solved so far indicated that conformational changes occur upon GTP hydrolysis. In EF-Tu, the conformational changes involve a large domain movement, as well as the concerted motions of two regions, called switch I and switch II [35, 42, 43]. Between the GMPPNP-bound and GDP-bound forms, the relative orientation of domain 1 to domains 2/3 drastically differs, but that between domains 2 and 3 is identical. Unlike EF-Tu, the archaeal aSelB [17] and aIF2γ [12] both undergo significant conformational changes only in switches I and/or II, and the relative orientations of domains 1 and 2/3 are retained between the GDP- and GMPPNP-bound forms.

In the present study, we determined the crystal structures of one of the *Methanosarcina* SelB/EF-Tu/aIF2γ-like proteins, MM1309 from *M. mazei,* in the GMPPNP-bound, GDP-bound, and apo forms, and found that the three structures shared similar conformations. The aminoacyl-binding pocket of MM1309 was too small to accommodate the pyrrolysyl moiety, contrary to the previous hypothesis for pyrrolysine incorporation [27, 28]. Interestingly, we discovered that MM1309 binds cysteinyl (Cys)-tRNA^Cys^, and slows its hydrolysis.

## Results and discussion

### MM1309 is a SelB/EF-Tu/aIF2γ-like protein

**Fig. 1:**
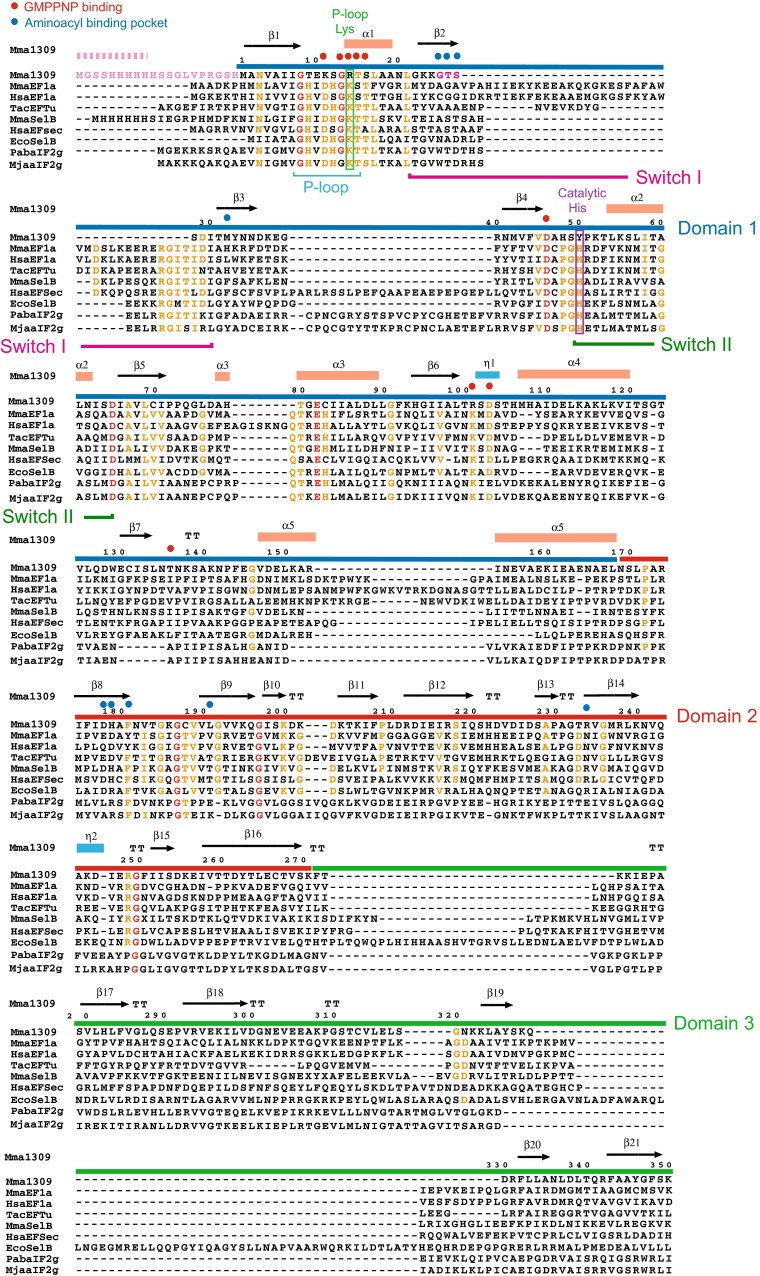
Structure-based sequence alignment of MM1309 with EF-Tu, EF1α, SelB, EF-Sec, and aIF2γ. The amino acid sequences were aligned using the programs CLUSTAL W [94] and ESPript [95], and then parts were optimized and adjusted manually. Completely and highly conserved amino acid residues are colored red and orange, respectively. The P-loop Lys residue, which interacts with the guanine nucleotide, and the catalytic His residue conserved among the GTPase family members are boxed in green and purple, respectively, on the sequence alignment. The secondary structures (α-helices, 3_10_-helices, and β-sheets) of MM1309 are shown as *light orange boxes*, *sky blue boxes*, and *black arrows*, respectively, on the *top line*. The MM1309 residues Lys11, Gly13, Arg14, Thr15, Ser16, Asp46, Arg103, Asp105, and Thr136, which interact with GMPPNP, are highlighted with *red circles* above the sequence alignment. The MM1309 residues Gly25, Thr26, Ser27, Met32, Met178, His179, and Leu191, which form the aminoacyl binding pocket, are highlighted with *blue circles* above the sequence alignment. The residues Gly25, Thr26, and Ser27, which are specific to MM1309, are *colored pink*. *Dashes* represent breaks in the actual amino acid sequences of the respective proteins, to allow sequence alignment with MM1309. The *numbers* at the *top* correspond to the amino acid residues of *M. mazei* MM1309. The hexahistidine tag derived from pET28 is *colored light pink*, and the disordered region (residues Met-20–His-11) of MM1309 is shown with a *light pink dotted line* above the sequence alignment. The P-loop (Gly8–Thr15), switch I (Gly22–Ile30), and switch II (Tyr50–Asp65) regions are underlined in *sky blue*, *pink*, and *green* below the sequence alignment. Mma1309, *M. mazei* MM1309 (AAM31005); MmaEF1a, *M. mazei* aEF1α (AAM31960); HsaEF1a, *Homo sapiens* EF1α (ABO30531); TacEFTu, *T. aquaticus* EF-Tu (CAA46998); MmaSelB, *M. maripaludis* aSelB (CAF30892); HsaEFSec, *Homo sapiens* EF-Sec (NP_068756); EcoSelB, *E. coli* SelB (AAC76614); PabaIF2 g, *P. abyssi* aIF2γ (Q9V1G0); MjaaIF2 g, *M. jannaschii* aIF2γ (Q58657)

The *M. mazei* genome encodes the general archaeal elongation factor aEF1α (422 residues) and MM1309 (350 residues) (Fig. 1) [44]. The MM1309 homologues are strictly conserved among the methanogenic archaeal genera *Methanosarcina*, *Methanococcoides*, *Methanohalophilus*, *Methanolobus*, *Methanomethyloborans*, *Methanocella*, *Methanosaeta*, *Methanococcus*, and *Methanocaldococcus*. The sequence identities of *M. mazei* MM1309 with *M. maripaludis* aSelB and *E. coli* SelB are 28 and 25 %, respectively, while those with *T. aquaticus* EF-Tu and *P. abyssi* aIF2γ are 21 and 23 %, respectively. Actually, a previous phylogenetic analysis revealed that MM1309 resembles SelB, rather than EF-Tu (EF1α) and aIF2γ [45].

### Overall structures of MM1309

**Fig. 2:**
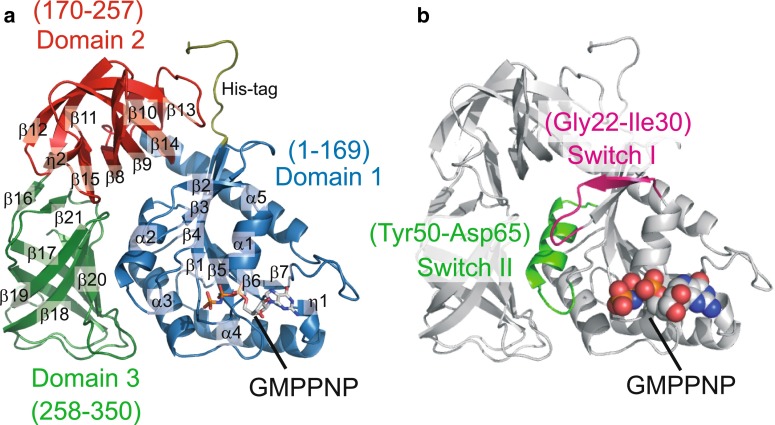
Structure of MM1309 bound with a GTP analogue. **a** Ribbon diagrams of MM1309. The bound GTP analog (GMPPNP) is shown as a stick model. Domains 1, 2, and 3 of MM1309 are *colored* *blue*, *red*, and *green*, respectively. Secondary structure assignments (α-helices, 3_10_-helices, and β-sheets) are shown as α, η, and β, respectively. **b** The switch I (Gly22–Ile30) and switch II (Tyr50–Asp65) motifs are *colored pink* and *green*, respectively. The GMPPNP molecule is shown as a space-filling model

**Fig. 3:**
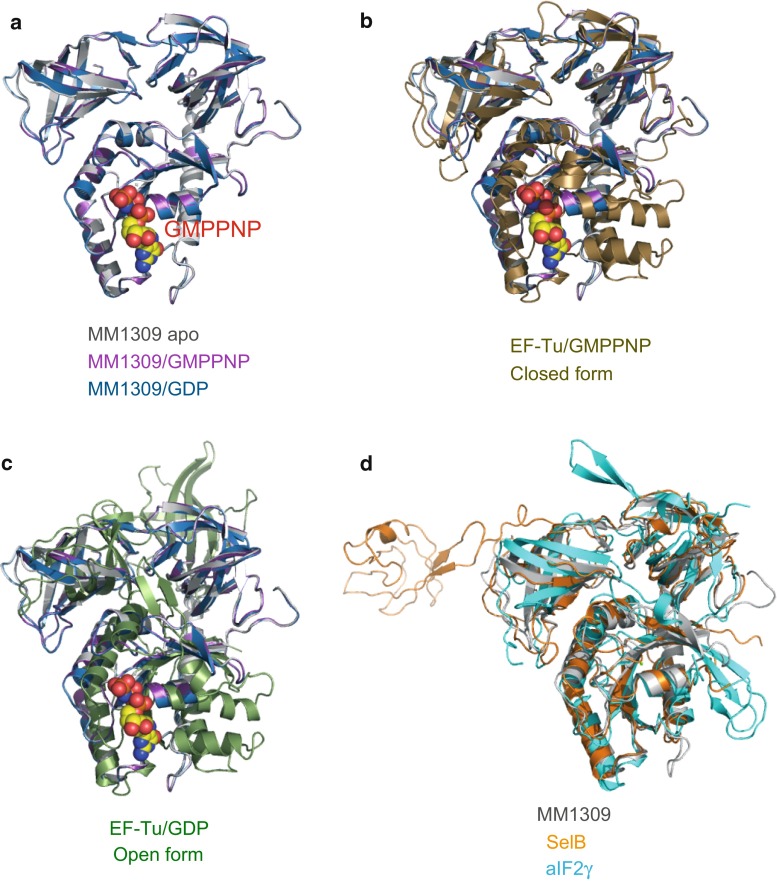
Superposition of MM1309 with EF-Tu, aSelB, and aIF2γ, represented by ribbon models. **a** Superposition of the MM1309 structures in the GMPPNP-bound, GDP-bound, and apo forms. **b** Superposition of MM1309 with *T. aquaticus* EF-Tu in the GTP-bound form (PDB code: 1TTT). **c** Superposition of MM1309 with *T. aquaticus* EF-Tu in the GDP-bound form (PDB code: 1TUI). **d** Superposition of MM1309 with *M. maripaludis* aSelB (PDB code: 4ACA) and with *P. abyssi* aIF2γ (PDB code: 1KK0)

We determined the crystal structures of *M. mazei* MM1309 in the GMPPNP-bound, GDP-bound, and apo forms at 1.7, 1.9, and 1.55-Å resolutions, respectively (“Materials and methods”, Table 1). The asymmetric unit contains one MM1309 molecule, and its 350 residues and the 11 tag-derived residues are all visible in the electron density map (Figs. 1, 2). The models show good geometry and all residues are in the allowed regions of the Ramachandran plot, as evaluated by Procheck [46] and Molprobity [47]. No significant structural differences were observed between these three forms, except for the nucleotide bound to the protein, as discussed below. The r.m.s.d. values between the three structures are less than 0.1 Å for 350 Cα atoms (Fig. 3). Hence, for the structure analysis in this study, the coordinates of the apo form, with the best resolution, were used unless otherwise noted.

**Table 1:** Data-collection and refinement statistics

	*M.mazei* MM1309 SeMet, GMPPNP-bound form	*M.mazei* MM1309 SeMet, GDP-bound form	*M.mazei* MM1309 Native, apo form
PDB code	3WNB	3WNC	3WND
X-ray source	PF BL5A	PF BL5A	SPring-8 BL41XU
Wavelength	0.9794	0.9794	1.0000
Space group	*P*2(1)2(1)2	*P*2(1)2(1)2	*P*2(1)2(1)2
Cell dimensions			
*a* (Å)	62.06	62.02	61.87
*b* (Å)	108.70	108.70	108.61
*c* (Å)	58.32	58.39	58.62
α, β, γ (°)	90, 90, 90	90,90,90	90, 90, 90
Resolution (Å)	50–1.6 (1.97–1.6)	50–1.9 (1.93–1.9)	50–1.55 (1.58–1.55)
I/σ (I)	34.49 (3.39)	14.4 (2.8)	16.48 (1.84)
Completeness (%)	100 (100)	99.0 (89.1)	98.1 (99.9)
No. reflections	52,887	31,405	57,020
Redundancy (%)	14.3	6.7	4.9
*R*_sym_^a^	9.2 (80.9)	3.7 (51.3)	8.9 (75.1)
Refinement			
*R*_work_^b^/*R*_free_ (%)^c^	18.9/23.3	17.2/21.7	17.3/20.9
Resolution (Å)	19.88–1.7	33.49–1.9	27.15–1.55
No. atoms			
Protein	2,765	2,765	2,815
Others	33	29	26
Water	400	389	307
No. reflections (work/test)	39,680/4,434	29,802/1,555	51,110/5,781
Average B-factors			
Protein	18.18	23.20	23.22
Ligands	44.72	59.25	64.20
Water	31.02	35.35	31.26
R.m.s deviations			
Bond length (Å)	0.008	0.009	0.015
Bond angles (°)	1.6	1.6	1.665
Ramachandran plot			
Most favored (%)	92.3	92.3	91.3
Allowed (%)	7.4	7.7	8.6
Disallowed (%)	0.3	0.0	0.0

The numbers in parentheses are for the last shell

^a^ *R*_sym_ = Σ|*I*_avg_ − *Ii·*|/Σ*Ii*

^b^ *R*_work_ = Σ|*F*_o_ − *F*_c_|/Σ *F*_o_ for reflections of work set

^c^ *R*_free_ = Σ|*F*_o_ − *F*_c_|/Σ *F*_o_ for reflections of test set [5–10 % of total reflections for *M.mazei* MM1309]

MM1309 consists of three structural domains (domains 1–3), a common feature in the members of the EF-Tu/SelB/aIF2γ superfamily (Figs. 1, 2). Domain 1 (residues 1–169) contains the nucleotide binding site, and consists of seven β strands surrounded by five α helices and one 3_10_ helix. Domain 2 (residues 170–257) and domain 3 (residues 258–350) are β barrel structures, consisting of nine and seven β strands, respectively. Domains 1 and 2 are connected with a long α helix (α5) in domain 1 and a short 3_10_ helix (η2) in domain 2. In contrast, domains 1 and 2 in the EF-Tu structure are connected by a loop, which corresponds to the hinge region for the large domain movement. MM1309 is in the closed domain conformation: domain 1 is packed onto domains 2 and 3, and adopts the same domain organization as that in the EF-Tu·GMPPNP complex (Figs. 2, 3a–c). The structure of the connecting region of MM1309 is much more rigid than that of EF-Tu, implying that the closed conformation is the most stable structure, and large domain movement upon nucleotide binding is unlikely. The closed domain arrangement has also been observed for SelB and aIF2γ (Fig. 3d) [12, 17]. A DALI search [http://www.embl-ebi.ac.uk/dali, 48] revealed that the structure of *M. mazei* MM1309 superimposed well on those of *M. maripaludis* aSelB (PDB codes: 4ACA, 4ACB, and 4AC9) [17], *Aeropyrum pernix* aEF1α (PDB codes: 3WXM and 3VMF) [49, 50], *S. solfataricus* aIF2γ (PDB codes: 2AHO, 3PEN, and 4M53) [12], *T. thermophilus* EF-Tu (PDB codes: 1EXM, 4LC0, 4LBV, 4LBY, 4LBZ, 4LBW, and 4H9G) [51], and *T. aquaticus* EF-Tu (PDB codes: 1EFT, 1B23, and 1TTT) [10, 31, 33], with Z-scores of 36.0–37.8, 36.8–37.4, 35.9–36.5, 35.9–36.2, and 35.8–36.1, respectively. The r.m.s.d. values between MM1309 and the EF-Tu/SelB/aIF2γ superfamily proteins are as follows: *M. maripaludis* aSelB·GMPPNP (4ACB, 2.6 Å for 340 Cα atoms) [17], *T. aquaticus* EF-Tu·GMPPNP·Phe-tRNA^Phe^ (1TTT, 2.5 Å for 336 Cα atoms) [31], *T. thermophilus* EF-Tu·GMPPNP (1EXM, 2.5 Å for 336 Cα atoms), *E. coli* EF-Tu·GMPPNP (2BVN, 2.8 Å for 340 Cα atoms) [52], *A. pernix* aEF1α·GTP·Pelota (3WXM, 2.2 Å for 334 Cα atoms) [49], *P. abyssi* aIF2γ (1KK2, 2.7 Å for 325 Cα atoms) [37], *S. solfataricus* aIF2γ·GDP (4M53, 2.4 Å for 331 Cα atoms), and *M. jannaschii* aIF2γ (1S0U, 2.7 Å for 314 Cα atoms) [38]. Thus, the closed form of MM1309 is not due to the crystal packing, but is the intrinsic structure of the protein.

### The guanine nucleotide binding site of MM1309

**Fig. 4:**
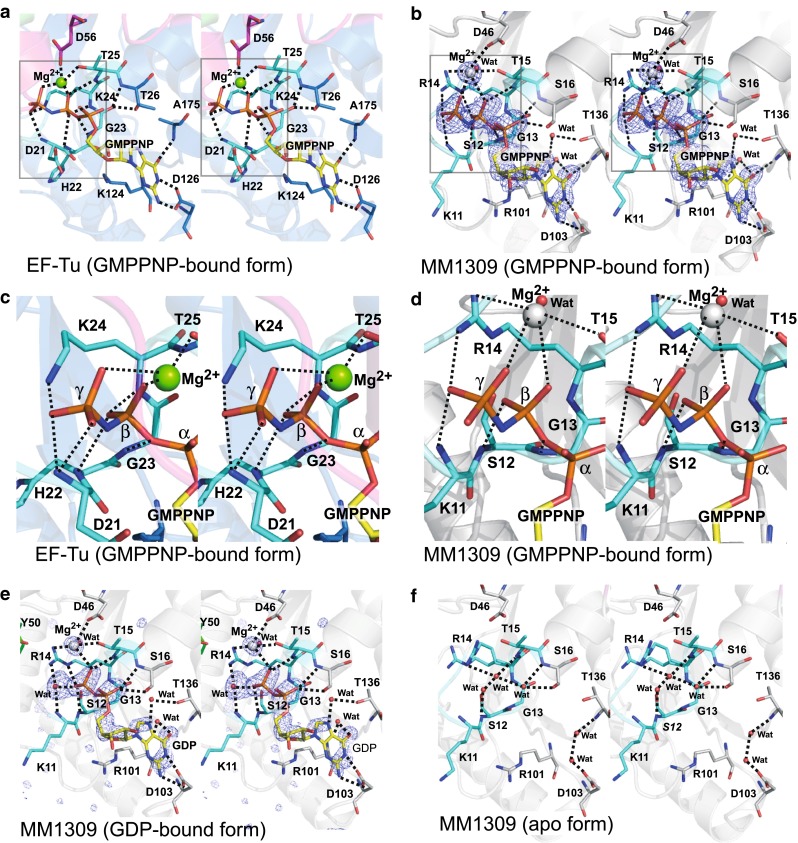
Stereo views of the GTP binding sites. **a** The bound GMPPNP molecule in the *T. aquaticus* EF-Tu·GMPPNP·Mg^2+^ structure. **b** The bound GMPPNP molecule in the MM1309·GMPPNP·Mg^2+^ structure. The *F*_o_–*F*_c_ omit map (contoured at 3.3 σ) of the bound GMPPNP·Mg^2+^ in the MM1309 active site. **c**, **d** Close-up stereo views around the γ-phosphate group of the bound GMPPNP in *T. aquaticus* EF-Tu·GMPPNP·Mg^2+^ (**c**) and MM1309·GMPPNP·Mg^2+^ (**d**). The amino acid residues surrounding the phosphate groups and the magnesium ions of the bound GMPPNP·Mg^2+^ are depicted by stick models. **e** The bound GDP molecule in the MM1309·GDP structure. The *F*_o_–*F*_c_ omit map (contoured at 4.0 σ) of the bound GDP·Mg^2+^ in the MM1309 active site. **f** The GTP binding site in the MM1309 apo form. The MM1309 residues that are located close to the bound guanine nucleotide are represented as stick models. The P-loop motifs (Gly17–Thr25 in EF-Tu and Gly7–Thr15 in MM1309) are shown in *sky blue*. The switch I regions are *colored pink*. Transparent ribbon models of EF-Tu (*blue*) and MM1309 (*white*) are visible in the background

The guanine nucleotide binding site in MM1309 is superimposable on those of the EF-Tu/SelB/aIF2γ superfamily proteins (Fig. 4). The electron density is well defined for the phosphate moiety and the guanine base, but is weaker for the ribose than the other moieties of GMPPNP. In the crystal of the MM1309·GMPPNP complex, the phosphate moiety is recognized by residues Lys11–Ser16 (corresponding to the EF-Tu residues His22–Thr26), which correspond to part of the P-loop (Fig. 4a–d) [53–55]. However, the highly-conserved Lys residue in the P-loop (GxxxxGK[S/T] where x can be any amino acid residue) is substituted with Arg (Arg14) in MM1309 (Fig. 1). Furthermore, the highly-conserved catalytic His residue of the Pro-Gly-His sequence in the GTPase family [56] is replaced by Tyr50 in MM1309 (Fig. 1). The main-chain nitrogen atoms of Lys11, Ser12, Gly13, Thr15, and Ser16 interact with the phosphate moiety. In addition, the side chain of Ser15 hydrogen bonds with one of the phosphate oxygen atoms. The amino group and the *N* ^ε^ atom of Arg14 hydrogen bond with the β- and γ-phosphate moieties, respectively (Fig. 4b, d). By contrast, in the case of the Ras-like GTPases, the side-chain amino group of the conserved Lys residue recognizes the β- and γ-phosphate moieties [55].

The guanine ring is mainly recognized by the conserved Asp103, located in the 3_10_ helix between β6 and α4 (Figs. 1, 4b). The side-chain carboxyl group of Asp103 hydrogen bonds with the N1- and N2-atoms of the guanine moiety. In addition, the main-chain nitrogen atoms of Thr136 and Arg101 hydrogen bond with the O6 of the guanine moiety, directly and via a water molecule, respectively. The side-chain oxygen atom of Thr136 also interacts with the N7 atom of the guanine moiety, via a water molecule. There is no specific interaction between the ribose moiety and MM1309. This may be one of the reasons why the electron density is weaker for the ribose, as compared to those for the guanine and phosphate moieties. The Mg^2+^ ion is mainly coordinated by the β- and γ-phosphate moieties (3.0 Å) and a water molecule (2.3 Å) (Fig. 4b, d). In addition, the side chain atoms of Thr15 (2.9 Å), Arg14 (3.4 Å), and Asp46 (3.3 Å) participate in the Mg^2+^ coordination. The *N* ^ε^ of Arg14 also interacts with the water molecule coordinating Mg^2+^. In the MM1309·GDP structure, the Mg^2+^ is coordinated by the five atoms in the same manner, except for the γ-phosphate moiety (Fig. 4e).

In the apo-form structure, there are three water molecules corresponding to the phosphate oxygen atoms, which form a hydrogen bonding network (Fig. 4f). These water molecules hydrogen bond with the side-chain guanidino group of Arg14, the main-chain nitrogen atoms (Lys11, Ser12, Thr15, and Ser16), and the side-chain oxygen atoms (Ser12 and Ser16), mimicking the interactions between the phosphate moieties and MM1309 in the GMPPNP-bound form. Regarding the guanine-binding site, the N1 and O6 atoms are replaced by water molecules, which hydrogen bond with Asp103 and Thr136. As a result, the conformations of the nucleotide-binding sites are the same in the three structures.

### The switch I and II motifs are involved in domain interactions, rather than nucleotide binding

**Fig. 5:**
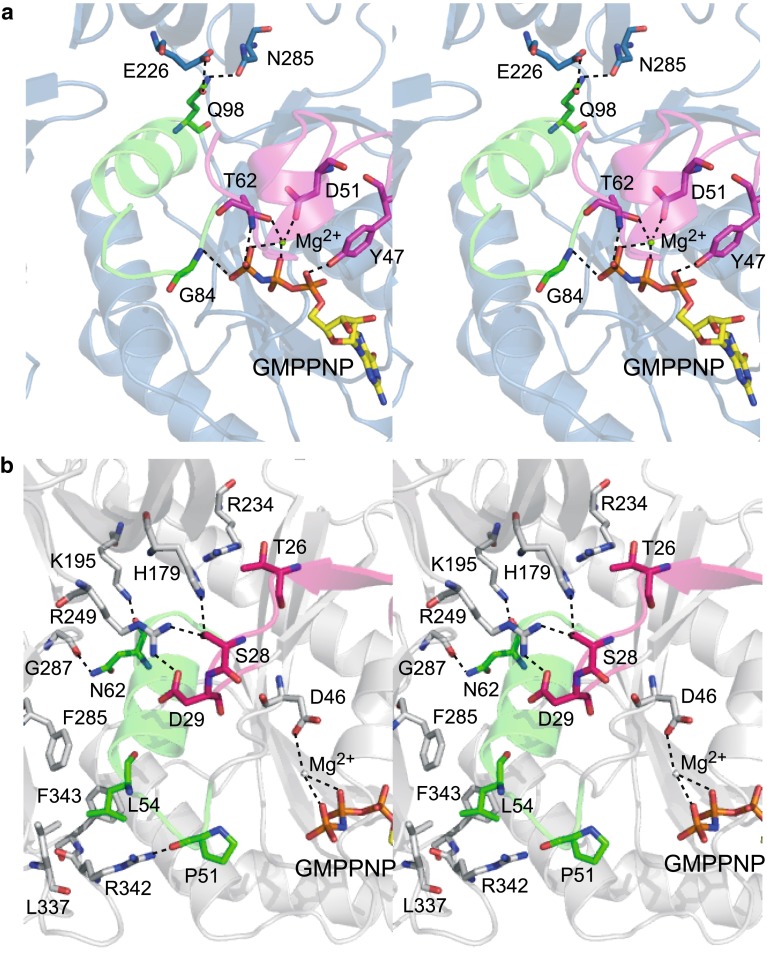
Close-up stereo views of the switch I and II regions in EF-Tu (**a**) and MM1309 (**b**). The bound GMPPNP molecule and the Mg^2+^ ion, and the EF-Tu and MM1309 residues in the switch I and II regions, which are involved in the GMPPNP interactions, are shown as stick models. The EF-Tu and MM1309 residues that are involved in the domain–domain interactions are also shown as stick models. The switch I and II regions of MM1309 are involved in domain–domain interactions, rather than GTP/GDP interactions. The switch I and II regions are *colored pink* and *green*, respectively. Transparent ribbon models of EF-Tu (*blue*) and MM1309 (*white*) are visible in the background

In many GTPases with solved structures of the GTP (GMPPNP)-bound, GDP-bound, and/or apo forms, significant conformational changes occur only in two regions, called “switch I” and “switch II” (Figs. 2, 5) [35, 42, 43]. In general, these regions interact with the phosphate moieties, and undergo conformational changes in the GTP hydrolysis cycle. For example, the structure of SelB in the GDP-bound form is very similar to that of the apo form, and differs only in the switch II region [17]. In aIF2γ, the structural change is limited to the switch I and II regions, among the GTP (GMPPNP)-bound, GDP-bound, and apo forms [39]. In contrast, both regions in MM1309 are primarily involved in domain–domain interactions, rather than interactions with the phosphate moieties (Figs. 2, 3, 5).

In EF-Tu, switch I (Thr32–Thr65) is located near the GTP binding site. The residues Tyr47, Asp51, and Thr62 in the switch I region interact with the GMPPNP phosphate moieties and the Mg^2+^ ion (Fig. 5a). Furthermore, the main-chain nitrogen atom of Gly84 in switch II (His85–Asp100) hydrogen bonds with the γ-phosphate moiety of GMPPNP. The switch II region is located near domains 1 and 2, but there are no interactions between the switch I region and domains 2/3, except for the hydrogen bonding interactions between Gln98 and Glu226/Asn285. In MM1309, the region corresponding to switch I (Gly22–Ile30) forms a β strand (β2) and is located far from the nucleotide binding site (Fig. 5b). Moreover, the switch I region is involved in the interaction between domains 1 and 2. The side chain of Thr26 interacts with that of Arg234 in domain 2, via a water molecule. The side chain of Ser28 hydrogen bonds with those of His179 and Arg249 in domain 2. The side chain of Arg249 also interacts with that of Asp29. These interactions may stabilize the relative orientation of domains 1 and 2. There is no direct interaction between the switch II region and GTP. The side chain of Asp46 interacts with the Mg^2+^ ion (Fig. 5b). Furthermore, part of the switch II region (Tyr50–Asp65) interacts with domains 2 and 3. The main-chain carbonyl group of Asn62 hydrogen bonds with the side chain of Lys195, while the side-chain amide group of Asn62 hydrogen bonds with the main-chain carbonyl group of Gly287. The main-chain carbonyl group of Pro51 hydrogen bonds with the side chain of Arg342. Leu54 forms van der Waals interactions with Phe285, Leu337, Arg342, and Phe343 in domain 3.

### MM1309 has higher affinity for GTP than GDP and GMPPNP

**Fig. 6:**
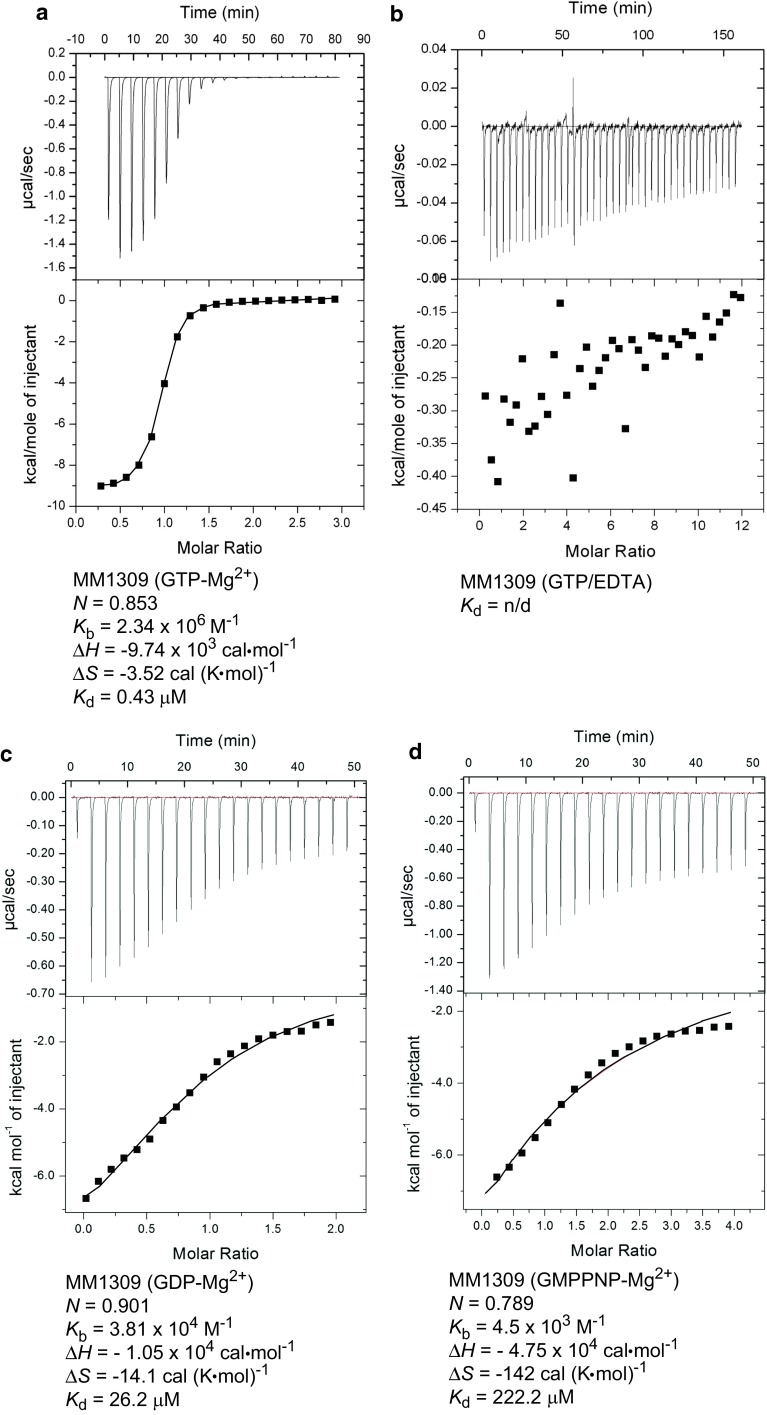
ITC analysis. The upper and lower panels display the ITC titration curves and the binding isotherms, respectively, for MM1309 with GTP·Mg^2+^ (**a**), GTP without Mg^2+^ (**b**), GDP·Mg^2+^ (**c**), and GMPPNP·Mg^2+^ (**d**). *N*, the binding stoichiometry; *K*_b_, the observed binding constant; *K*_d_ (*K*_d_ = 1/*K*_b_), the dissociation constant; ∆*H*, the binding enthalpy; ∆*S*, the binding entropy

The GTP- and GDP-bound forms of the translational GTPases including EF-Tu and SelB, regulate translation initiation, elongation, and termination on the ribosome [57]. We examined the affinities of MM1309 for GTP, GDP, and GMPPNP in the presence of Mg^2+^ ions, and GTP in the absence of Mg^2+^ ions, by isothermal titration calorimetry (ITC) (Fig. 6). MM1309 bound GTP·Mg^2+^ with a dissociation constant (*K*_d_) of 0.43 μM (Fig. 6a), while that for GTP without Mg^2+^ could not be determined (Fig. 6b). On the other hand, MM1309 bound GDP·Mg^2+^ weakly, with a dissociation constant (*K*_d_) of 26.2 μM (Fig. 6c). In general, EF-Tu binds GDP much more strongly than GTP (*K*_d_^GTP^, 0.375 μM; *K*_d_^GDP^, 0.0013 μM) [58], while SelB binds GTP more strongly than GDP (*K*_d_^GTP^, 0.7 μM; *K*_d_^GDP^, 13.4 μM) [59]. The *K*_d_ values of MM1309 for GTP and GDP are similar to those of SelB, rather than those of EF-Tu. These results indicated that, like SelB, MM1309 does not need a guanine nucleotide exchange factor (GEF). Surprisingly, MM1309 bound GMPPNP·Mg^2+^ much less strongly than GTP·Mg^2+^, with a dissociation constant (*K*_d_) of 222.2 μM (Fig. 6d). MM1309 did not hydrolyze GTP during the ITC analysis. We examined whether MM1309 has intrinsic GTPase activity in the absence of ribosomes by using radioactively-labeled [α-^32^P]GTP and a fluorescent GTP analog, [2′-/3′-*O*-(*N*-methylanthraniloyl)guanosine-5′-*O*-triphosphate] (Mant-GTP), but did not detect any GTPase activity (data not shown). Therefore, MM1309 lacks GTPase activity, at least in the absence of ribosomes. These results are supported by the fact that the highly conserved P-loop Lys and catalytic His residues in the GTPase family are replaced by Arg14 and Tyr50, respectively, in MM1309 (Fig. 1).

Notably, the binding affinity of MM1309 for GMPPNP was 500 times lower than that for GTP (Fig. 6d). Therefore, the present GMPPNP-bound structure, which is very simlar to the GDP-bound structure, may be different from the true GTP-bound structure. In this context, the structural properties of the GTPase translation factors are diverse [60–80]. First, eukaryotic release factor 3 (eRF3) in complex with GMPPNP undergoes large conformational changes in the presence of eukaryotic release factor 1 (eRF1) and the ribosome [68–73], while eRF3 exhibits about 300 times lower affinity for GMPPNP than GTP in the presence of eRF1. In contrast, SelB displays similar affinities for GTP and GMPPNP, although its overall structures may differ between them [80]. However, EF-Tu undergoes large changes in the switch region conformations and the domain arrangement upon GMPPNP binding, whereas the conformation of elongation factor G (EF-G)·GMPPNP is the same as that of EF-G·GDP, but drastically changes upon ribosome binding [60–69]. Therefore, we should further investigate the true GTP-bound form and the GTPase activity of MM1309.

### Docking models of MM1309 with aminoacyl-tRNAs

**Fig. 7:**
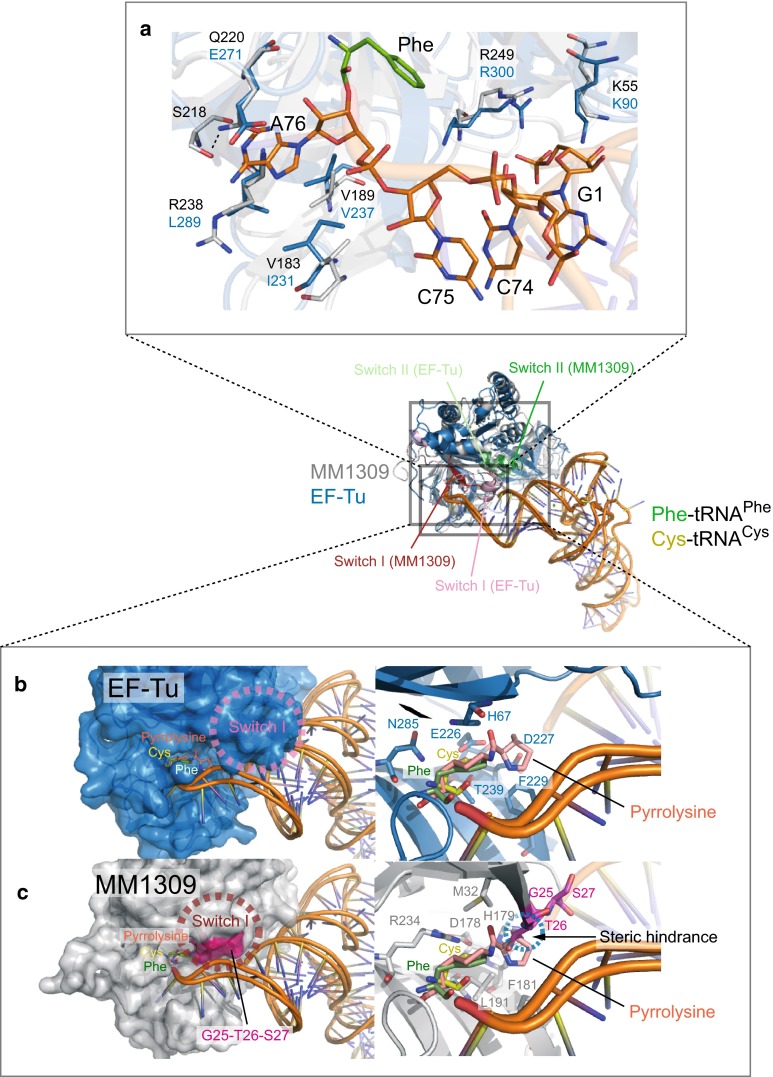
Docking model of MM1309 with EF-Tu·Phe-tRNA^Phe^ and EF-Tu·Cys-tRNA^Cys^. **a** Superimposition of the 5′-A and 3′-CCA tRNA binding site residues (shown as stick models) in MM1309 on those in EF-Tu·Phe-tRNA^Phe^. **b**, **c** Comparison of the aminoacyl binding sites between MM1309 and EF-Tu. The MM1309 (*grey*) and EF-Tu (*marine blue*) residues superimposed well on each other. EF-Tu and MM1309 are represented as surface models, and tRNAs are represented as ribbon models. The modeled pyrrolysyl moiety is also shown as a stick model. In contrast to the aminoacyl binding pocket of EF-Tu, the MM1309 pocket lacks sufficient space to accommodate the pyrrolysyl moiety, because of the steric hindrance with Gly25, Thr26, and Ser27 in β2

The structure of MM1309 superimposed well on those of the *T. aquaticus* EF-Tu·GMPPNP·Phe-tRNA^Phe^ (PDB code: 1TTT) and EF-Tu·GMPPNP·Cys-tRNA^Cys^ (PDB code: 1B23) ternary complexes (Figs. 3b, 7) [31, 33]. The 3′-end of the tRNA resides in a hydrophobic pocket composed of the side chains of Ile231, Val237, Leu289, and Glu271 in EF-Tu, which correspond to Val183, Val189, Arg238, and the Gln220 side chain in MM1309, respectively (Fig. 7a). However, the direction of the Gln220 side chain differs from that of Glu271 in the EF-Tu complex. Glu220 hydrogen bonds with the side chain of Ser218, which causes steric hindrance between MM1309 and the adenine base of the modeled tRNA (Fig. 7a). Therefore, Gln220 may undergo a conformational change upon tRNA binding, in order to accommodate A76 in the binding pocket. By contrast, the binding site for the 5′-end of the tRNA is blocked by the interdomain interaction, although the residues involved in the tRNA binding are well conserved between MM1309 and EF-Tu. In the EF-Tu ternary complex structure, Lys90 and Arg300, which respectively correspond to Lys55 and Arg249 in MM1309, are directly involved in the 5′ phosphate recognition (Fig. 7a). In MM1309, the aforementioned interdomain contacts may prevent the tRNA binding. Therefore, the residues should undergo conformational changes in order to interact with tRNA, which may rearrange the switch I and II conformations. A slight movement of the switch I region could be sufficient to accommodate the 5′-end of the tRNA, as judged by a comparison between the EF-Tu and MM1309 structures. The bottom of the aminoacyl binding pocket of EF-Tu, which is composed of His67, Glu226, Asp227, Phe229, Thr239, and Asn285, has sufficient space to accommodate the pyrrolysyl moiety (Fig. 7b). In contrast, the aminoacyl binding pocket of MM1309, which is composed of Gly25, Thr26, Ser27, Met32, His170, Asp178, Phe181, Leu191, and Arg234, is narrow and lacks space for the pyrrolysyl moiety (Fig. 7c). The MM1309 residues Gly25, Thr26, and Ser27 in β2, which are involved in the tRNA binding site, cause especially severe steric hindrance with the docked pyrrolysyl moiety (Fig. 7c).

### The phylogenetic distributions of the MM1309 orthologues are different from those of the pyrrolysine, selenocysteine, and phosphoserine incorporation systems

**Fig. 8:**
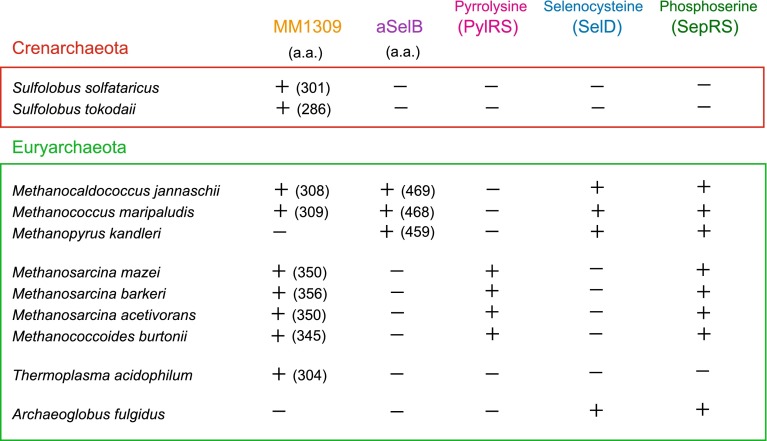
The phylogenetic distribution of MM1309 orthologues is unrelated to those of the pyrrolysine, selenocysteine, and phosphoserine systems. A plus or minus sign indicates whether the genes encoding MM1309-like proteins, aSelB, PylRS, SelD, and SepRS, exist in archaea. The numbers of amino acid residues (a.a.) in the MM1309-like proteins and aSelBs are also shown

A previous phylogenetic analysis revealed that the existence of the MM1309 proteins in archaea has no relevance to the presence of the pyrrolysine and selenocysteine incorporation systems [45]. Among archaea, a pyrrolysine-related protein (PylRS) exists only in *Methanosarcinaceae*. On the other hand, selenocysteine-related proteins (SelB and SelD) exist only in *Methanocaldococcaceae*, and *Methanococcaceae*, but not in *Methanosarcinaceae*, *Sulfolobaceae*, and *Thermoplasmataceae* (Fig. 8). Furthermore, a phosphoserine-related protein [phosphoseryl-tRNA synthetase (SepRS)] exists in *Methanocaldococcaceae*, *Methanococcaceae*, *Methanosarcinaceae*, and *Archaeoglobaceae*, but not in *Sulfolobaceae* and *Thermoplasmataceae*, indicating that the phosphoserine system is also unrelated to the phylogenetic distribution of the MM1309 orthologues (Fig. 8). Regardless of the presence of the pyrrolysine, selenocysteine, and phosphoserine systems, the MM1309 genes might have been horizontally transferred among several archaea. Atkinson et al. [45] proposed that MM1309 binds Cys-RNA^Cys^ and protects the cysteinyl moiety from oxidation, after they examined the initial version of our MM1309 structure in the Protein Data Bank (PDB code: 2ELF) and considered that MM1309 could accommodate the cysteinyl moiety in the aminoacyl binding pocket (Fig. 7c). Furthermore, the MM1309 proteins are conserved among anaerobic archaea. Anaerobic archaea might retain a similar strategy for cysteine protection, considering that the structural models for the aminoacyl sites of the MM1309 proteins from *S. solfataricus*, *M. jannaschii*, and *T. acidophilum* closely resemble that of MM1309 (data not shown).

### MM1309 binds Cys-tRNA^Cys^

**Fig. 9:**
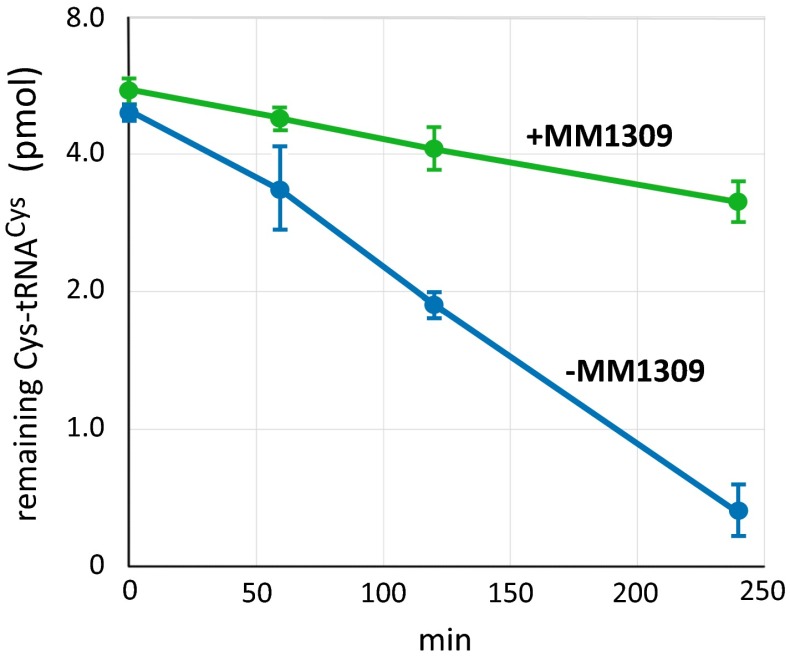
MM1309 binds Cys-tRNA^Cys^ and slows its hydrolysis. The reaction mixture was prepared without MM1309 and Cys-tRNA^Cys^, and then Cys-tRNA^Cys^ or Cys-tRNA^Cys^ preincubated with MM1309 was added to each tube. The reactions were stopped at after 0, 1, 2, and 4 h by adding 10 % trichloroacetic acid (TCA), and were washed several times with TCA. Then, the radioactivities were measured with a liquid scintillation counter. The *vertical axis* is plotted on a logarithmic scale. Cys-tRNA^Cys^ in the presence of MM1309 (*green line*) exhibited slower deacylation than that without MM1309 (*blue line*). The half-lives of Cys-tRNA^Cys^ with and without MM1309 were about 300 and 80 min, respectively. *Error bars* SD (n = 3)

Based on the hypothesis described above, we examined if MM1309 binds Cys-tRNA^Cys^ (Fig. 9). We prepared radioactively-labeled Cys-tRNA^Cys^ by using cysteinyl-tRNA synthetase (CysRS) and tRNA^Cys^ from *M. mazei* [81], and performed an aminoacyl-tRNA hydrolysis protection assay according to the standard method [82]. In the absence of MM1309, [^14^C]Cys-tRNA^Cys^ was hydrolyzed with a half-life of 80 min (Fig. 9, blue line). On the other hand, the half-life of hydrolysis was much longer (300 min) in the presence of MM1309 (Fig. 9, green line), indicating that MM1309 binds Cys-tRNA^Cys^ and slows its hydrolysis.

What is the physiological role of MM1309 in *M. mazei* cells? As MM1309 homologues are conserved among many anaerobic archaea, it may be reasonable that MM1309 protects Cys-tRNA^Cys^ as a guardian in the oxidative environment. It is also possible that MM1309 acts as an alternative translation elongation factor, for the following two reasons. First, MM1309 might be able to accommodate the 20 canonical amino acids in the aminoacyl-binding pocket, based on the docking model. Second, *M. mazei* aEF1α, which contains 19 cysteine residues, might be prone to oxidation. On the other hand, MM1309 contains only 6 cysteine residues, and the purified protein remains soluble even under oxidative conditions. Thus, we propose the designation of MM1309, as well as its orthologues, as aEF-X, toward further investigations of the physiological roles of the aEF-X protein in *M. mazei* cells.

## Materials and methods

### Materials, enzymes, and chemicals

Biochemical and molecular biological procedures were performed using commercially available enzymes, chemicals, and other materials. GTP, GDP, and guanosine 5′-(β,γ-imido)triphosphate (GMPPNP) were purchased from Sigma-Aldrich (USA). [2′-/3′-*O*-(*N*-methylanthraniloyl)guanosine-5′-*O*-triphosphate] (Mant-GTP) and [2′-/3′-*O*-(*N*-methylanthraniloyl)guanosine-5′-*O*-diphosphate] (Mant-GDP) were purchased from Jena Bioscience (Germany). [α-^32^P]GTP (800 Ci/mmol) and [^14^C] l-cystine (74 GBq/mmol) were purchased from Perkin Elmer (USA).

### Cloning, expression, and purification of *M. mazei* MM1309

The *M. mazei* MM1309 gene was cloned into the pET28c vector (Novagen). The native and selenomethionine (SeMet)-substituted proteins were overexpressed in *E. coli* BL21(DE3) and B834(DE3) cells, respectively. The cell pellet was resuspended and sonicated in 50 mM potassium phosphate buffer (pH 7.4), containing 10 mM imidazole, 500 mM NaCl, 5 mM β-mercaptoethanol, 10 % glycerol, and protease inhibitor cocktail (Complete-EDTA free, Roche) (buffer A). After centrifugation, the supernatant was loaded on a HisTrap column (GE Healthcare), and the protein was eluted with buffer A containing 500 mM imidazole, instead of 10 mM imidazole. Fractions containing the MM1309 protein were pooled and dialyzed against 50 mM potassium phosphate buffer (pH 7.4), containing 50 mM NaCl, 1 mM DTT, 10 % glycerol, and protease inhibitor cocktail (buffer B). The dialyzed fraction was then loaded on a Resource Q column (GE Healthcare), and the flow-through fraction was applied to a hydroxyapatite column (BioRad). After washing the column with buffer B, the bound proteins were eluted by a linear gradient of 0.05–0.83 M NaCl. The proteins were dialyzed against buffer B, and then loaded onto a HiTrap heparin column (GE Healthcare). After washing the column with buffer B, the proteins were eluted by a linear gradient of 0.05–0.83 M NaCl. Prior to crystallization, the MM1309 protein fraction was dialyzed against 10 mM Tris–HCl buffer (pH 8.0), containing 150 mM NaCl, 10 mM MgCl_2_, and 10 mM β-mercaptoethanol, and concentrated to 12.1–15.3 mg/ml using an Amicon 15 filter (Millipore).

### Crystallization

The MM1309 protein was crystallized by the hanging-drop vapor-diffusion method, at 20 °C. The initial screening of crystallization conditions was conducted using commercially available screening kits. The crystals used for data collection were obtained by mixing 1 μl of protein solution with 1 μl of reservoir solution. The reservoir solution contained 0.1 M sodium acetate buffer (pH 4.4–4.8) and 1.4 M sodium citrate. Plate-shaped crystals grew to dimensions of 0.2 mm × 0.1 mm × 0.04 mm in a day. To obtain the co-crystals of MM1309 with GMPPNP or GDP, the MM1309 protein was crystallized in the presence of 5 mM nucleotide in the crystallization drop. The co-crystals were harvested with a solution containing 5 mM GMPPNP or GDP.

### Data collection, structure determination, and refinement

The single-wavelength anomalous dispersion (SAD) data sets from the SeMet derivative protein co-crystals with GMPPNP or GDP were collected at beamline BL5A of the Photon Factory (Tsukuba, Japan). The data set of the native protein was collected at beamline BL41XU of SPring-8 (Harima, Japan). All data were processed using the HKL2000 program suite [83]. The MM1309 crystals belong to the orthorhombic space group *P*2_1_2_1_2, with unit cell dimensions of *a* = 62.06, *b* = 108.7, *c* = 58.32 Å, and the asymmetric unit contains one MM1309 molecule. The selenium sites were identified using SnB [84] with the SeMet/GMPPNP data set. The selenium sites were refined and the initial phases were calculated with SOLVE [85]. The phases were improved with density modification, using RESOLVE [85]. The initial model was automatically built by RESOLVE and ArpWarp [86], and was manually refined using O [87], CueMol [http://cuemol.sourceforge.ge.jp/en], and Coot [88]. The atomic model was refined using CNS [89], REFMAC5 [90], and PHENIX [91]. The models showed good stereochemistry and geometry, as analyzed by the programs Procheck [46] and Molprobity [http://molprobity.biochem.duke.edu/, 47]. The structures of the GDP-bound and apo forms were solved by the molecular replacement method, using Molrep [46] with the GMPPNP-bound form model as the search model, and refined in the same manner as the GMPPNP-bound form. Graphical images were prepared with the program PyMOL [http://pymol.sourceforge.net/]. All data collection and refinement statistics are summarized in Table 1. Superimpositions of the Cα traces of the MM1309 structures were produced by the program secondary structure matching (SSM) [92].

### Isothermal titration calorimetry (ITC)

ITC experiments were performed with the VP-ITC and auto auto-iTC200 systems (MicroCal, USA). In the calorimeter cell, 25–50 μM MM1309, in 10 mM Tris–HCl buffer (pH 7.5) containing 150 mM NaCl, 5 mM MgCl_2_, and 10 mM β-mercaptoethanol, was titrated with 1 mM GTP, 0.5 mM GDP, or 1 mM GMPPNP at 25 °C. Aliquots (2–5 μl) of ligands were injected into the 0.4–2-ml cell containing the MM1309 solution, to achieve a complete binding isotherm. The resulting titration curves were fitted using the MicroCal Origin software. The binding constant (*K*_b_), the binding stoichiometry (*N*), and the enthalpy variations (∆*H*) were determined by a nonlinear regression fitting procedure.

### Preparation of Cys-tRNA^Cys^

The *M. mazei* tRNA^Cys^ (5′-GCCAAGGUGGCGGAGCGGUCACGCAAUCGCCAGCAGAGCGAUUCAGUCCUGGUUCAAAUCCGGACCUUGGCUCCA-3′) transcript was prepared by in vitro transcription, according to the standard protocol [93]. Briefly, the transcription reaction was performed at 37 °C for 4 h, in a reaction mixture (5 ml) containing 80 mM Hepes–NaOH buffer (pH 8.1), 20 mM MgCl_2_, 40 mM KCl, 20 mM dithiothreitol (DTT), 2 mM spermine, 14 μg/ml bovine serum albumin (BSA), 20 mM GMP, 5 mM each of ATP, GTP, CTP and UTP, 0.28 mg/ml T7 RNA polymerase, 5 unit pyrophosphatase (Sigma), 0.5 μl ribonuclease inhibitor (TOYOBO), and 10 μg/ml PCR-amplified DNA as a template. The products were purified by Resource Q column chromatography. The tRNA^Cys^ transcript used in this study was charged with cysteine using *M. mazei* CysRS [58]. The *M. mazei* CysRS gene was amplified from genomic DNA, and cloned into the pET28 vector. *M. mazei* CysRS was overexpressed in *E. coli* BL21-Gold(DE3) (Agilent Technologies), and purified by two column chromatography steps (HisTrap and Resource Q). The CysRS fractions were dialyzed against 20 mM potassium phosphate buffer (pH 7.4), containing 0.15 M NaCl and 5 mM β-ME. The aminoacylation reaction was performed at 37 °C for 20 min, in a reaction mixture containing 40 mM Tris–HCl buffer (pH 7.5), 20 mM MgCl_2_, 40 mM KCl, 4 mM ATP, 50 mM DTT, 40 μM [^14^C]-L-cystine (74 GBq/mmol), 5 μM *M. mazei* CysRS, and 10 μM *M. mazei* tRNA^Cys^. The Cys-tRNA^Cys^ was purified by ethanol precipitation, and finally dissolved in 6 mM potassium acetate (pH 5.0) to a concentration of 2 μM. The concentration of Cys-tRNA^Cys^ was estimated from the labeled amino acids incorporated within the tRNA.

### Deacylation assay

The assay was basically performed as previously described [59]. Briefly, the deacylation reaction mixture contained 50 mM Tris–HCl buffer (pH 8.5), 20 mM KCl, 25 mM NaCl, 7 mM MgCl_2_, 1 mM DTT, 1 mM GTP, and 4.5 μM Cys-tRNA^Cys^, with or without 33 μM MM1309. The Cys-tRNA^Cys^ was preincubated with or without MM1309 at 30 °C for 10 min, and then the deacylation assay buffer was added. The deacylation reaction was performed at 25 °C for 4 h.

### Data deposition

The atomic coordinates and structure factors for the apo form of MM1309, and the GMPPNP- and GDP-bound forms of SeMet-substituted MM1309 from *M. mazei*, have been deposited in the Protein Data Bank (PDB codes: 3WND, 3WNB, and 3WNC, respectively).

## Abbreviations

EF-Tu: Translation elongation factor Tu
EF-Sec: The selenocysteine tRNA-specific elongation factor or SelB
a/eIF2γ: Archaeal/eukaryotic initiation factor 2 gamma
PylRS: Pyrrolysyl-tRNA synthetase
SAD: Single-wavelength anomalous dispersion
SeMet: Selenomethionine
GMPPNP: Guanosine 5′-(β,γ-imido)triphosphate
ITC: Isothermal titration calorimetry
r.m.s.d.: Root mean square deviation
